# Comparing the effects of ipragliflozin versus metformin on visceral fat reduction and metabolic dysfunction in Japanese patients with type 2 diabetes treated with sitagliptin: A prospective, multicentre, open‐label, blinded‐endpoint, randomized controlled study (PRIME‐V study)

**DOI:** 10.1111/dom.13750

**Published:** 2019-05-08

**Authors:** Masaya Koshizaka, Ko Ishikawa, Ryoichi Ishibashi, Yoshiro Maezawa, Kenichi Sakamoto, Daigaku Uchida, Susumu Nakamura, Masaya Yamaga, Hidetaka Yokoh, Akina Kobayashi, Shunichiro Onishi, Kazuki Kobayashi, Jun Ogino, Naotake Hashimoto, Hirotake Tokuyama, Fumio Shimada, Emi Ohara, Takahiro Ishikawa, Mayumi Shoji, Shintaro Ide, Kana Ide, Yusuke Baba, Akiko Hattori, Takumi Kitamoto, Takuro Horikoshi, Ryota Shimofusa, Sho Takahashi, Kengo Nagashima, Yasunori Sato, Minoru Takemoto, Laura Kristin Newby, Koutaro Yokote, On Behalf of the PRIME‐V Study Group

**Affiliations:** ^1^ Department of Diabetes, Metabolism and Endocrinology Chiba University Hospital Chiba Japan; ^2^ Department of Endocrinology, Hematology and Gerontology Chiba University Graduate School of Medicine Chiba Japan; ^3^ Division of Diabetes, Endocrinology and Metabolism Kimitsu Chuo Hospital Chiba Japan; ^4^ Department of Internal Medicine, Hotaruno Central Naika Chiba Japan; ^5^ Department of Internal Medicine, Odayama Clinic Chiba Japan; ^6^ Department of Diabetes and Metabolism Japanese Red Cross Narita Hospital Chiba Japan; ^7^ Department of Diabetes and Metabolism Asahi General Hospital Chiba Japan; ^8^ Department of Diabetes, Endocrine and Metabolic Diseases Tokyo Women's Medical University Yachiyo Medical Center Chiba Japan; ^9^ Department of Internal Medicine, Yukarigaoka Tokuyama Medical Clinic Chiba Japan; ^10^ Department of Diabetes and Metabolism National Hospital Organization Chiba Medical Center Chiba Japan; ^11^ Geriatric Medical Center Chiba University Hospital Chiba Japan; ^12^ Diagnostic Radiology and Radiation Oncology Chiba University Graduate School of Medicine Chiba Japan; ^13^ Department of Radiology Sannou Hospital Chiba Japan; ^14^ Clinical Research Center Chiba University Hospital Chiba Japan; ^15^ Department of Global Clinical Research Chiba University, Graduate School of Medicine Chiba Japan; ^16^ Department of Diabetes, Metabolism and Endocrinology, School of Medicine, International University of Health and Welfare, Chiba, Japan; ^17^ Duke Clinical Research Institute Duke University Medical Center Durham North Carolina

**Keywords:** insulin sensitivity, ipragliflozin, metformin, sitagliptin, visceral fat

## Abstract

A prospective, multicentre, open‐label, blinded‐endpoint, randomized controlled study was conducted to evaluate the efficacy of treatment with ipragliflozin (sodium‐dependent glucose transporter‐2 inhibitor) versus metformin for visceral fat reduction and glycaemic control among Japanese patients with type 2 diabetes treated with sitagliptin, HbA1c levels of 7%‐10%, and body mass index (BMI) ≥ 22 kg/m^2^. Patients were randomly assigned (1:1) to receive ipragliflozin 50 mg or metformin 1000‐1500 mg daily. The primary outcome was change in visceral fat area as measured by computed tomography after 24 weeks of therapy. The secondary outcomes were effects on glucose metabolism and lipid metabolism. Mean percentage reduction in visceral fat area was significantly greater in the ipragliflozin group than in the metformin group (−12.06% vs. −3.65%, *P* = 0.040). Ipragliflozin also significantly reduced BMI, subcutaneous fat area, waist circumference, fasting insulin, and homeostatic model assessment (HOMA)‐resistance, and increased HDL‐cholesterol levels. Metformin significantly reduced HbA1c and LDL‐cholesterol levels and increased HOMA‐beta. There were no severe adverse events. The use of ipragliflozin or metformin in combination with dipeptidyl peptidase‐4 inhibitors, widely used in Japan, may have beneficial effects in ameliorating multiple cardiovascular risk factors.

## 1 INTRODUCTION

Visceral fat accumulation has been shown to correlate with metabolic abnormalities.^1^ While metformin is established as the first‐choice medication for patients with type 2 diabetes (T2D) in the United States and Europe, 60% of patients on oral antidiabetic drugs receive dipeptidyl peptidase‐4 inhibitors (DPP‐4is) as the first choice in Japan.^2^ DPP‐4is have been widely used in East Asia, where patients with T2D are characterized primarily by beta‐cell dysfunction, less obese, and have higher insulin sensitivity compared with Caucasians.^3^ Indeed, DPP‐4is reportedly show greater glucose‐lowering effects among East Asians.^4^

For Japanese patients initially treated with DPP‐4is who failed to achieve optimal glycaemic control, metformin is a candidate as the second‐line medication. A combination of metformin and DPP‐4i reportedly reduces body weight.^5^ Sodium‐dependent glucose transporter‐2 inhibitors (SGLT2is) decrease blood glucose level in a non‐insulin‐dependent manner, and reportedly reduce body weight.^6^ Randomized clinical trials have suggested that SGLT2is may have an effect of reducing cardiovascular events.^7^ However, it is still unclear how SGLT2is improve metabolic dysfunction. In previous studies, SGLT2is have been administered as the second‐line drug to patients already receiving metformin. The pathophysiology and common treatment regimen for T2D in Japanese patients differs from previous studies; therefore, the present study was designed to address these nuances in the treatment of Japanese patients.

To clarify the second‐line antidiabetes drug that is preferable for Japanese patients with T2D after DPP‐4i, this study investigated the effect of ipragliflozin, a novel SGLT2i, on visceral fat accumulation, as measured by computed tomography (CT), compared with the effect of metformin as the second drug for patients already receiving sitagliptin.

## 2 METHODS

This was a prospective, multicentre, open‐label, blinded‐endpoint, randomized controlled study. The design has been described previously.^8^ The protocol was approved by the responsible ethics committees and was conducted in full compliance with the Declaration of Helsinki. Participants provided written informed consent and were enrolled from September 2014 to September 2016. The study was registered at http://www.umin.ac.jp/ctr/ (UMIN‐ID: UMIN 000015170).

Eligible participants were diagnosed with T2D, were aged 20‐75 years old, had received DPP‐4i (sitagliptin 50 mg daily) for ≥12 weeks, and had current HbA1c > 7.0% and < 10.0% and body mass index (BMI) > 22.0 kg/m^2^.^9^ The exclusion criteria are described in the Appendix S1 (see the supporting information for this article).

Participants were randomly assigned to the ipragliflozin or metformin group in a 1:1 allocation. Patients in the ipragliflozin group received oral ipragliflozin 50 mg daily. Patients in the metformin group were initially administered 500 mg of metformin daily, and then 1000 mg daily after 2 to 4 weeks. For the patients with an inadequate glucose‐lowering effect observed in the metformin group, the dose of metformin was increased to 1500 mg daily at 12 weeks. During the study period, diet, exercise therapy, and other drugs did not change, as orally verified by the physicians.

The primary outcome was any change in the visceral fat area in 24 weeks between the two groups. CT imaging was performed before study drug administration and after 24 weeks. Two radiologists, who were masked to patients' clinical information and treatment assignment, centrally evaluated the CT images. Secondary outcomes included changes in HbA1c, body weight and BMI, waist circumference, fasting plasma glucose and insulin levels, homeostatic model assessment (HOMA)‐beta, HOMA‐R, total cholesterol, LDL‐cholesterol, fasting triglycerides, and HDL‐cholesterol, blood pressure, adiponectin, high sensitivity C‐reactive protein (hs‐CRP), subcutaneous and total fat area. Safety was assessed by recording all adverse events that were observed during the study.

In the primary analysis, the least‐square mean difference in any change in the visceral fat area in 24 weeks between the two groups and its 95% confidence intervals (CI) were estimated using ANCOVA adjusted for age, baseline waist circumference, HbA1c, and baseline visceral fat area. All *P*‐values are two‐sided. *P* < 0.05 was considered statistically significant.

## 3 RESULTS

The enrolment process is presented in Figure S1. Participant characteristics were balanced between the groups (Table S1). In the metformin group, five (10.4%) patients continued with 500 mg daily dose while for 24 (49.0%) and 19 (38.8%) patients the daily dose increased to 1000 and 1500 mg, respectively. The average dosage of metformin was 1124 mg.

### 3.1 Body composition

**Figure 1:**
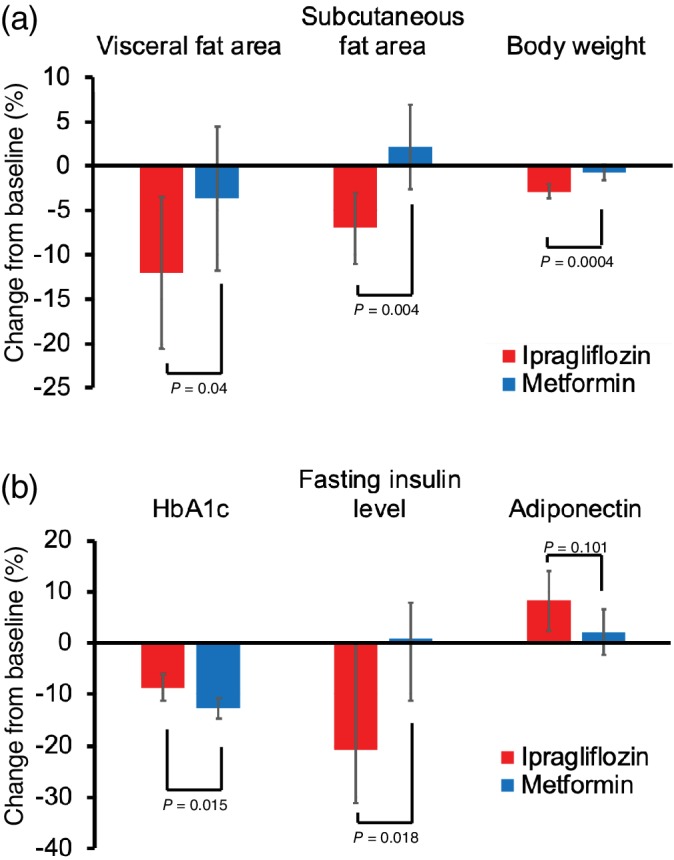
Change from baseline in visceral fat area, subcutaneous fat area (upper right), body weight, HbA1c, fasting insulin level, and adiponectin after 24 weeks of treatment. Coloured columns show mean values and black bars show 95% confidence intervals

Figures 1 and S2 and Tables 1 and S2 show the primary and secondary outcome results. The reduction in visceral fat area in the ipragliflozin group was significantly greater than in the metformin group [−12.06% vs. −3.65%; group difference (95% CI) ‐8.40%, (−16.4 to −3.38), *P* = 0.040] (Figure 1). The reduction in total and subcutaneous fat areas, body weight, BMI, and waist circumference were significantly greater in the ipragliflozin group than in the metformin group (Figure 1, Table 1).

**Table 1:** Primary and secondary outcomes at 24 weeks

	Ipragliflozin n = 48	Metformin n = 50	Difference between groups		
	Change from baseline (%)	Change from baseline (%)	Change from baseline (%)	95% CI	*P*‐value
Body composition					
Visceral fat area	−12.06	−3.65	−8.40	−16.43, −3.38	0.040
Subcutaneous fat area	−7.03	2.15	−9.18	−15.34, −3.03	0.004
Total fat area	−7.98	0.37	−8.35	−13.98, −2.72	0.004
Body weight	−2.88	−0.74	−2.15	−3.31, −0.98	0.0004
BMI	−2.88	−0.74	−2.15	−3.31, −0.98	0.0004
Waist circumference	−2.85	−0.37	−2.48	−3.92, −1.03	0.001
Glycaemic control					
HbA1c	−8.70	−12.73	4.03	0.79, 7.27	0.015
Fasting plasma glucose	−12.22	−14.15	1.93	−4.47, 8.33	0.551
Fasting insulin level^a^	−20.73	0.85	−18.56	−34.20, −2.80	0.018
HOMA‐beta^a^	9.05	26.04	−22.51	−37.79, −2.18	0.029
HOMA‐R^a^	−25.25	0.00	−17.08	−32.86, −1.91	0.024
Blood lipid panels					
Total cholesterol	1.65	−5.94	7.60	3.07, 12.12	0.001
Triglyceride^a^	−14.46	0.86	−11.49	−24.92, 4.23	0.165
LDL‐cholesterol	3.06	−7.57	10.63	2.81, 18.44	0.008
HDL‐cholesterol	8.74	1.51	7.22	2.10, 12.34	0.006
Other assessments					
Systolic blood pressure	−2.98	−2.19	−0.79	−4.99, 3.41	0.710
Diastolic blood pressure	−2.93	0.61	−3.54	−8.88, 1.81	0.192
Adiponectin	8.33	2.20	6.13	−1.22, 13.47	0.101
hs‐CRP^a^	−10.18	−18.75	7.48	−17.89, 34.62	0.590

*Note:* Changes at 24 weeks from baseline are shown as means unless otherwise indicated.

Abbreviations: BMI, body mass index; CI, confidence interval; HOMA, homeostatic model assessment; hs‐CRP, high sensitivity C‐reactive protein.

a The data were not normally distributed and had outliers; non‐parametric analysis (Wilcoxon rank sum test and group difference confidence interval by Hodges‐Lehmann estimator) was performed. Changes from baseline are shown as median.

### 3.2 Glycaemic control

Both drugs reduced HbA1c and fasting plasma glucose levels. The relative reduction in HbA1c level was greater in the metformin group than in the ipragliflozin group at 24 weeks (−12.73% vs. ‐8.70%, *P* = 0.015) (Figure 1). By contrast, the relative reduction in fasting insulin level in the ipragliflozin group was significantly greater than in the metformin group, in which there was an increase at 24 weeks (−20.73% vs. 0.85%, *P* = 0.018) (Figure 1). The increase in HOMA‐beta was significantly greater in the metformin group than in the ipragliflozin group (26.04% vs. 9.05%, *P* = 0.029). The decrease in HOMA‐R was significantly greater in the ipragliflozin group than in the metformin group, in which there was no change (−25.25% vs. 0.00%, *P* = 0.024).

### 3.3 Blood lipid panels

Metformin significantly reduced total and LDL‐cholesterol levels compared with ipragliflozin, in which there was an increase (−5.94% vs. 1.65%, *P* = 0.001; −7.57% vs. 3.06%, *P* = 0.008, respectively) at 24 weeks. By contrast, the reduction in triglycerides was significantly greater in the ipragliflozin group compared with the metformin group, in which there was an increase (−12.72 vs. 11.69, *P* = 0.006) at 8 weeks. The increase in HDL‐cholesterol was significantly greater in the ipragliflozin group than in the metformin group (8.74% vs. 1.51%, *P* = 0.006) at 24 weeks (Figure S2).

### 3.4 Other assessments

Changes in adiponectin, hs‐CRP and blood pressure were similar between the two groups (Figure S3).

### 3.5 Adverse events

Ipragliflozin showed significantly less gastrointestinal disturbances and more thirst and frequent urination compared with metformin. There were no severe adverse events (Table S3).

## 4 DISCUSSION

Compared with metformin, ipragliflozin significantly reduced the visceral fat area when used as a secondary agent in combination with DPP‐4i. Ipragliflozin also reduced body weight, BMI, subcutaneous fat area, waist circumference, fasting insulin, and HOMA‐R and triglyceride levels, and increased HDL‐cholesterol levels. By contrast, metformin significantly reduced HbA1c and LDL‐cholesterol levels and significantly increased HOMA‐beta compared with ipragliflozin. Although HbA1c decreased less in the ipragliflozin group compared with the metformin group, the decrease in visceral fat in the ipragliflozin group was greater than that in the metformin group.

Visceral fat accumulation is associated with insulin resistance and various metabolic complications.^1^ In this study, in which evaluation was based on blinded CT image measurement, administration of ipragliflozin resulted in reduced visceral fat area, even in combination with a DPP‐4i, an insulin secretagogue; by contrast, metformin combined with a DPP‐4i did not affect visceral fat area. Metformin primarily affects the liver, prevents gluconeogenesis, and does not reduce total glucose amount in the body. Therefore, its effect on fat mass reduction was insufficient. Ipragliflozin reduces glucose re‐absorption in the proximal renal tubules, and the subsequent reductions in glucose availability require an alternative fuel source. SGLT2is reportedly increase fatty acid oxidation, fat utilization, browning, and lipolysis in white adipose tissue.^10^ This mechanism might have mediated the visceral fat reduction observed in this study.

Waist circumference percentage reductions were smaller than those in the visceral fat area. This could indicate that waist circumference, which was used as a surrogate measure of central adiposity, underestimates visceral fat. The visceral fat reduction in the ipragliflozin group could be partially a result of the water reduction in adipose tissue. However, the slight reduction in blood pressure by ipragliflozin may indicate that a dehydration effect of ipragliflozin was not particularly strong. In the present study, the visceral fat area was measured by use of CT, which has been reported to be highly correlated with total visceral adipose tissue mass with significance.^11^

This study showed that ipragliflozin simultaneously reduced visceral fat and fasting insulin in association with elevation of adiponectin levels from the baseline. Fat loss increases adiponectin,^12^ which regulates glucose levels and fatty acid breakdown and reverses insulin resistance.^13^ In the ipragliflozin group, adiponectin levels increased from baseline, a result which may have been associated with reduced fasting insulin levels and reduced HOMA‐R, a marker of insulin resistance in the present study.

The extent to which HbA1c levels were reduced was greater in the metformin versus ipragliflozin group at 24 weeks. The combination of sitagliptin and metformin reportedly augments GLP‐1 secretion,^14^ which might have contributed to this result. Moreover, metformin increased HOMA‐beta, a marker of pancreatic insulin secretion, suggesting that the combination of metformin with DPP‐4i is suitable for Japanese patients with low insulin secretion and without excess visceral fat.

Clustering of multiple risk factors can remarkably increase the risk of atherosclerotic cardiovascular disease (ASCVD), even when the involved risk factors have mild individual impacts. Therefore, it is important to manage lipids and fasting glucose levels to efficiently prevent ASCVD. In the present study, both drugs had positive effects on improving dyslipidaemia. Ipragliflozin was associated with increased HDL‐cholesterol and decreased triglyceride levels. It is assumed that a decrease in visceral adiposity leads to a reduction of free fatty acid influx into the liver, which results in lower production of very low‐density lipoprotein (VLDL) triglyceride.^15^ Moreover, increased insulin sensitivity may improve lipoprotein lipase activity,^16^ facilitating VLDL‐triacylglycerol hydrolysis and HDL maturation.^17^ However, ipragliflozin increased LDL‐cholesterol. Previous studies have shown that SGLT2is increase LDL‐cholesterol levels.^7^, ^18^ In fasting conditions, SGLT2 inhibition switches from carbohydrate to fat oxidation and stimulates ketone body production and hepatic cholesterol synthesis. These metabolic alterations result in lower LDL receptor expression and moderate increases in LDL‐cholesterol levels.^19^ By contrast, metformin lowered LDL‐cholesterol levels, through increased recycling of LDL receptors via reduced arachidonic acid in the liver.^20^ The decrease in LDL‐cholesterol was observed in parallel with a decrease in total cholesterol. There were no significant differences in changes in blood pressure between the two groups.

This study has a few limitations. First, it was an open‐label trial; however, evaluators of the primary outcome were blinded to the clinical information. Second, the study population was small and limited to Japanese patients, which may affect the generalizability of the findings. Finally, the study period was limited to 24 weeks.

In conclusion, both drugs showed beneficial effects in ameliorating multiple cardiovascular risk factors. Ipragliflozin was superior in terms of reduced visceral fat, improved hyperinsulinaemia, and low HDL‐cholesterol. Metformin showed advantage in terms of improved hyperglycaemia and high LDL‐cholesterol. Metformin is used globally as the first‐line medication for T2D. To the best of our knowledge, the present study is the first to compare metformin and ipragliflozin as a secondary drug with a DPP‐4i. The study results may provide a rationale for alternative treatment strategies for T2D, especially in Japanese patients.

## CONFLICT OF INTEREST

K.Y. received research grants from Astellas Pharma Inc. and MSD K.K. (Tokyo, Japan). He also received a lecture fee from Astellas Pharma Inc. and Sumitomo Dainippon Pharma (Tokyo, Japan). No conflicts of interest are declared for the other authors.

## AUTHOR CONTRIBUTIONS

All authors made significant contributions to the study. K.Y. designed the original concept. MK and KI wrote the manuscript and managed the project. K.Y. and K.N. reviewed and edited the manuscript. K.I., M.K., T.I., K.K., and M.T. wrote the protocol. K.I., M.K., R.I., Y.M., K.S., D.U., S.N., M.Y., H.Y., A.K., S.O., K.K., J.O., N.H., H.T., F.S., E.O., TI, M.S., S.I., K.I., Y.B. recruited the patients and carried out physical examinations, including taking blood samples. T.H. and R.S. evaluated C.T. slices. S.T., K.N., and Y.S. performed the statistical analyses. All authors read the final manuscript and provided approval for the publication of the manuscript. K.Y. is the guarantor of this work and, as such, had full access to all the data in the study and takes responsibility for the integrity of the data and the accuracy of the data analysis.

## Supporting information


**Appendix S1** Supporting Information.

**Appendix S2** Supporting Information.
