# Supplementary material for: Comparing the effects of ipragliflozin versus metformin on visceral fat reduction and metabolic dysfunction in Japanese patients with type 2 diabetes treated with sitagliptin: A prospective, multicentre, open‐label, blinded‐endpoint, randomized controlled study (PRIME‐V study)
Source: Diabetes Obes Metab. 2019 May 8;21(8):1990–5. doi: 10.1111/dom.13750 (PMC6767075; doi:10.1111/dom.13750)
Supplement: Supplementary file 1 — Appendix S1 Supporting Information. [file DOM-21-1990-s001.docx]

**Comparing the effects of ipragliflozin versus metformin on visceral fat reduction and metabolic dysfunction in Japanese patients with type 2 diabetes treated with sitagliptin: A prospective, multicentre, open-label, blinded-endpoint, randomised controlled study (PRIME-V study)**

**Supplementary Appendix**

**Supplementary Methods**

**Inclusion and exclusion criteria**

Individuals with the following criteria were eligible: diagnosed with type 2 diabetes according to the diabetes diagnostic criteria;^1^ aged 20-75 years; had received DPP-4i (sitagliptin 50 mg daily) for ≥12 weeks; had current HbA1c >7.0% and <10.0% and current BMI >22.0 kg./m^2^; had estimated glomerular filtration rate >50.0 ml/min/1.73 m^2^; and understood this study and provided written informed consent. Patients with the following criteria were excluded: type 1 diabetes; history of diabetic ketoacidosis, diabetic coma or pre-coma within 6 months prior to the date of consent; serious infections, surgery, or serious trauma requiring insulin therapy; moderate or high renal dysfunction (male serum creatinine level [Cre] ≥1.3 mg/dL, female Cre ≥1.2 mg/dL); haemodialysis treatment (including peritoneal haemodialysis); severe liver injury; history of serious vascular complications (stroke, myocardial infarction, and heart failure) requiring hospital admission; administration of glucose-lowering agents other than sitagliptin; women who were pregnant, lactating, possibly pregnant, or planning pregnancy; history of hypersensitivity to DPP-4 inhibitor, SGLT2 inhibitor, or metformin; presence or possibility of urinary tract infection or dehydration, positive urinary ketone body; history of lactic acidosis; excessive alcohol intake; history of fracture due to osteoporosis; CT examination conducted within 3 months prior to consent date; or study deemed inappropriate for the participant by their doctor.

**Randomisation and blinding**

Eligible participants, enrolled by physicians, were patient-registered by facsimile in the registration centre at the Chiba University Clinical Trial Data Centre. Registration depended on three allocation factors simultaneously (i.e., age [<65 or ≥65 years], waist circumference [male <85 or ≥85 cm and female <80 or ≥80 cm], and HbA1c [<8.0% or ≥8.0% at baseline]). Participants were randomly assigned to the ipragliflozin or metformin group in a 1:1 allocation.

A minimisation method with biased-coin assignment using the data management system (ACReSS [Fujitsu, Tokyo, Japan]) computer program located at the registration centre was used. The computer program was concealed from researchers.

**Procedures**

Patients in the ipragliflozin group received oral ipragliflozin 50 mg daily, for 24 weeks. Patients in the metformin group were initially administered 500 mg of metformin daily, and then 1000 mg daily after two to four weeks, for 24 weeks (unless gastrointestinal disturbances occurred). With inadequate glucose-lowering effect in the metformin group, as evidenced by: a) HbA1c ≥7.4% at 12 weeks in patients with HbA1c ≥8.0% at baseline and b) HbA1c ≥6.9% at 12 weeks in patients with HbA1c <8.0% at baseline, the dose of metformin was increased to 1500 mg daily. During the study period, diet, exercise therapy, and other drugs remained the same as before the study initiation. No change in dosage or withdrawal of antiplatelet, antihypertensive, lipid lowering agents, or diuretics occurred during the study period. Physical examinations and clinical laboratory tests were conducted, and medication adherence was obtained at baseline, 2, 4, 8, 12, and 24 weeks. CT imaging for visceral fat area measurement was performed before ipragliflozin or metformin administration and after 24 weeks of administration. Conditions observed for conventional CT imaging were as follows: X-ray voltage and exposure were 120 kVp and 200 mAs, imaging position was at the fourth lumbar vertebra level, and imaging was performed at the end of expiration. The CT images were centrally evaluated by two independent radiologists, who were masked to patients’ clinical information. The data were analysed by FatScan (East Japan Institute of Technology, Ibaraki, Japan) to quantify the visceral, subcutaneous, and total fat areas; data on waist circumference were also collected.

**Statistical analysis**

The sample size, determined as 106 patients (53 patients in each group) was set with a difference of visceral fat area ratio of 0.17, standard deviation (SD) of 0.249, two-sided two-sample t-test at 0.05 level of significance, dropout rate of 10%, and 90% power.

The statistical analysis and reporting of this study were conducted in accordance with the CONSORT guidelines, with the primary analysis based on the full analysis set. For the baseline variables, summary statistics employed frequencies and proportions for categorical data, and mean and SD for continuous variables. Baseline variables were compared using chi-square or Fisher’s exact test for categorical outcomes and unpaired t-tests for continuous variables, as appropriate. In the primary analysis, the baseline-adjusted means and 95% confidence interval (CI) estimated by ANCOVA with the change in the visceral fat area at 24 weeks were compared between the ipragliflozin and metformin groups. The comparisons were adjusted for age, baseline waist circumference and HbA1c, and baseline visceral fat area. Secondary outcomes were intended to supplement the primary outcome; therefore, adjustment for multiplicity was not performed. The safety analysis was performed by Fisher’s exact test. All *P* values were two-sided. *P* values <0.05 was considered statistically significant. All analyses were performed by SAS Version 9.4 (SAS Institute, Cary, North Carolina, USA). Chief investigators and statisticians at Chiba University developed all statistical analysis plans that were finalised before database locking. A data monitoring committee oversaw the study.

**Supplementary Results**

In total, 103 patients were enrolled and randomly assigned to either the metformin (52 patients) or ipragliflozin (51 patients) group. During the follow-up, one patient in the ipragliflozin group withdrew due to diarrhoea (Figure S1).

Body Composition

The reduction in the total and subcutaneous fat areas at week 24 were both significantly greater in the ipragliflozin group than the metformin group (total fat area -7.98% vs. 0.37%, group difference [95% CI] -8.35% [-13.98 to -2.72], *P*=0.004; subcutaneous fat area -7.03% vs. 2.15%, group difference -9.18% [-15.34 to -3.03], *P*=0.004) (Figure 1, Table 1).

Adverse Events

More gastrointestinal disturbances, i.e., diarrhoea, loose stool, and constipation, were reported in the metformin group (26 [53.1%] patients) than in the ipragliflozin group (seven [14.3%] patients, *P*<0.0001). Thirst and frequent urination were more frequently reported in the ipragliflozin group. There were no significant differences between the two groups regarding other adverse events including hypoglycaemia, dehydration, urinary tract infection, and drug-induced skin eruption (Table S3).

**Acknowledgements**

The authors would like to thank the staff and patients who participated in the present study.

**Funding**

To conduct this study, an agreement was signed between Chiba University and Astellas Pharma Inc. (Tokyo, Japan), who funded this work. The funding source had no role in the design of this study; its execution, analyses, interpretation of the data, and decision to publish; or the preparation of the manuscript. The corresponding author had full access to all the data in the study and had final responsibility for the decision to submit for publication.

**Conflict of interest statement**

KY received research grants from Astellas Pharma Inc. and MSD K.K. (Tokyo, Japan). He also received a lecture fee from Astellas Pharma Inc. and Sumitomo Dainippon Pharma (Tokyo, Japan). No conflicts of interest are declared for other authors.

**Contribution statement**

All authors made significant contributions to the study. KY designed the original concept. MK and KI wrote the manuscript and managed the project. KY and KN reviewed and edited the manuscript. KI, MK, TI, KK, and MT wrote the protocol. KI, MK, RI, YM, KS, DU, SN, MY, HY, AK, SO, KK, JO, NH, HT, FS, EO, TI, MS, SI, KI, YB recruited the patients and carried out physical examinations, including taking blood samples. TH and RS evaluated CT slices. ST, KN, and YS performed the statistical analyses. All authors read the final manuscript and provided approval for the publication of the manuscript. KY is the guarantor of this work and, as such, had full access to all the data in the study and takes responsibility for the integrity of the data and the accuracy of the data analysis.

**Supplementary References**

1. Seino Y, Nanjo K, Tajima N, et al. Report of the Committee on the classification and diagnostic criteria of diabetes mellitus. Diabetol Int 2010;1:2-20.

**Supplementary Figure legends**

Figure. S1. Trial profile

Figure S2. Change from baseline in LDL-cholesterol (left), HDL-cholesterol (middle), and triglycerides (right), after 24 weeks of treatment with ipragliflozin or metformin. Coloured columns show mean values and black bars show 95% CIs.

Figure S3. Change from baseline in systolic blood pressure (left) and systolic blood pressure (right) after 24 weeks of treatment with ipragliflozin or metformin. Coloured columns show mean values and black bars show 95% CIs.

Figure S4. The interaction between reduction in visceral fat area and other possible affecting factors is shown as a forest plot of a subgroup analysis.

**Supplementary Figures**

Figure S1

Analysed (n= 48)

Analysed (n= 50)

Excluded (n= 0)

♦  Did not meet inclusion criteria (n= 0)

♦  Declined to participate (n= 0)

## Analysis

Allocated to ipragliflozin (n= 51)

♦ Received allocated intervention (n= 48)

♦ Did not receive allocated intervention

[Withdrew consent after randomisation] (n= 3)

## Follow-Up

## Allocation

Lost to follow-up (n= 0)

Discontinued intervention (n= 0)

Allocated to metformin (n= 52)

♦ Received allocated intervention (n= 50)

♦ Did not receive allocated intervention

[Withdrew consent after randomisation] (n= 2)

Randomised (n= 103)

Assessed for eligibility (n= 103)

## Enrollment

Lost to follow-up (n= 0)

Discontinued intervention

[due to diarrhoea] (n= 1)

Figure S2


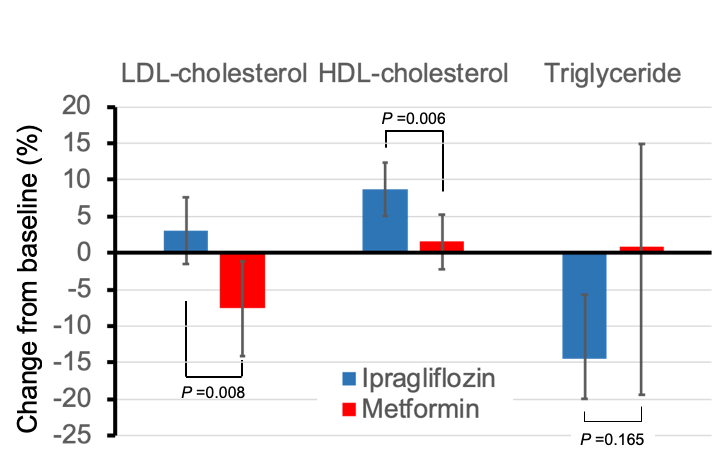


Figure S3

**
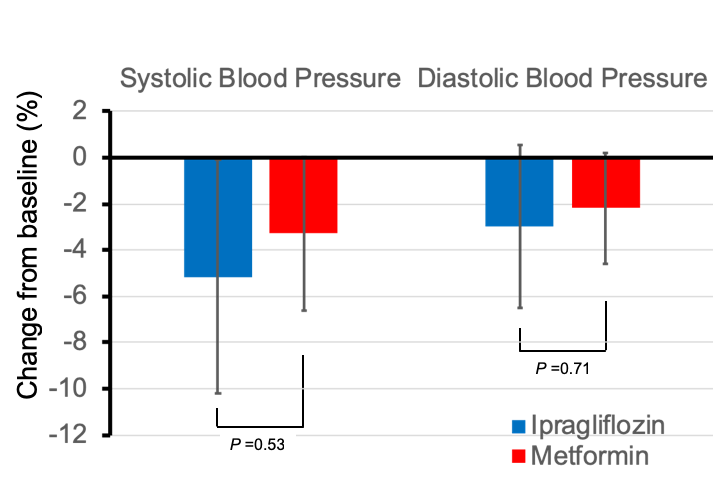
**

Figure S4

**
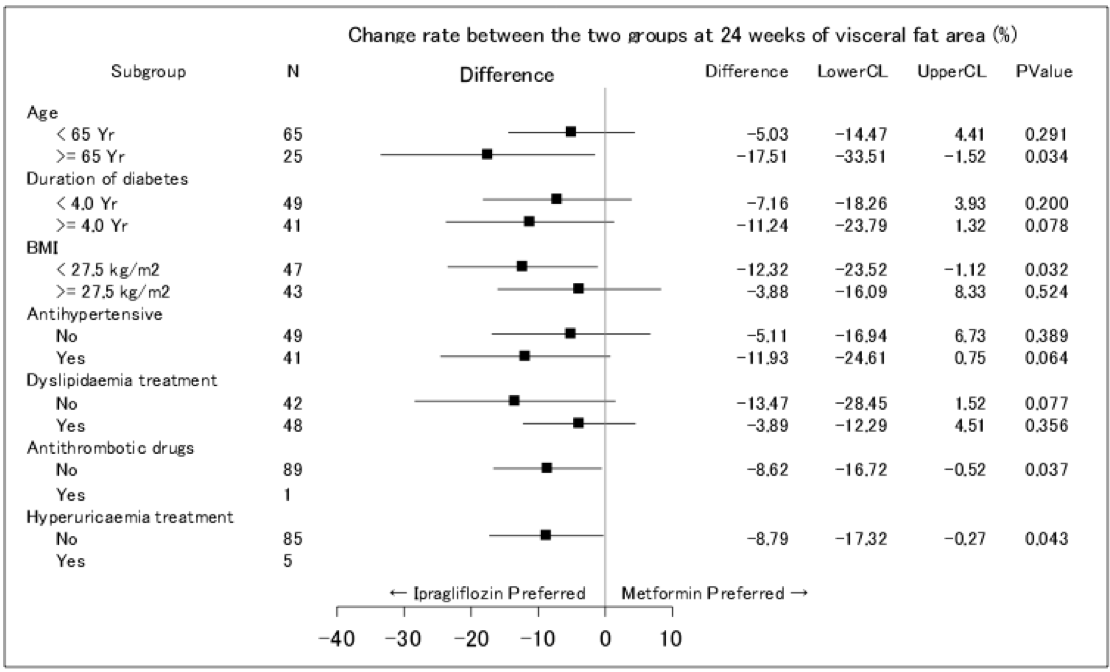
**

**Supplementary Tables**

Table S1 Baseline clinical characteristics

|  | Ipragliflozin group | | | Metformin group | | |  |
| --- | --- | --- | --- | --- | --- | --- | --- |
|  | n=48 | | | n=50 | | | *P*-value |
| Age (years) | 56.6 | ± | 11.9 | 55.7 | ± | 12.2 | 0.709 |
| Male, n (%) | 31 (64.6) | | | 28 (56.0) | | | 0.386 |
| Body weight (kg) | 73.08 | ± | 14.18 | 78.28 | ± | 18.37 | 0.121 |
| BMI (kg/m^2^) | 27.55 | ± | 4.24 | 28.83 | ± | 5.32 | 0.192 |
| Waist circumference (cm) | 93.19 | ± | 9.67 | 96.74 | ± | 12.28 | 0.124 |
| Duration of diabetes (years) | 5.4 | ± | 4.6 | 5.3 | ± | 4.8 | 0.937 |
| HbA1c (%) | 7.95 | ± | 0.73 | 8.12 | ± | 0.90 | 0.324 |
| Fasting plasma glucose (mg/dL) | 159.9 | ± | 35.8 | 166.1 | ± | 29.8 | 0.360 |
| Fasting insulin (μU/mL) | 10.62 | ± | 9.41 | 9.20 | ± | 6.77 | 0.418 |
| Total cholesterol (mg/dL) | 185.4 | ± | 29.3 | 194.6 | ± | 34.3 | 0.166 |
| Triglyceride (mg/dL) | 141.1 | ± | 69.5 | 162.5 | ± | 197.5 | 0.480 |
| LDL-cholesterol (mg/dL) | 106.6 | ± | 24.2 | 110.9 | ± | 27.2 | 0.412 |
| HDL-cholesterol (mg/dL) | 50.5 | ± | 11.0 | 52.3 | ± | 12.0 | 0.434 |
| Systolic blood pressure (mmHg) | 136.2 | ± | 17.8 | 133.8 | ± | 15.5 | 0.489 |
| Diastolic blood pressure (mmHg) | 82.1 | ± | 11.1 | 79.7 | ± | 11.4 | 0.300 |
| Adiponectin (μg/mL) | 7.37 | ± | 4.06 | 7.84 | ± | 3.69 | 0.568 |
| hs-CRP (mg/dL) | 0.126 | ± | 0.129 | 0.146 | ± | 0.138 | 0.475 |
| Visceral fat area (cm^2^) | 148.23 | ± | 67.89 | 162.51 | ± | 70.42 | 0.318 |
| Subcutaneous fat area (cm^2^) | 194.57 | ± | 81.13 | 220.06 | ± | 105.47 | 0.192 |
| Total fat area (cm^2^) | 342.80 | ± | 126.51 | 382.57 | ± | 144.06 | 0.157 |
| Drinking alcohol, n (%) | 28 (58.3) | | | 27 (54.0) | | | 0.666 |
| Smoking, n (%) | 20 (41.7) | | | 22 (44.4) | | | 0.816 |
| Past history |  | | |  | | |  |
| Cerebral infarction, n (%) | 0 (0) | | | 0 (0) | | | 1.000 |
| Cerebral haemorrhage, n (%) | 0 (0) | | | 1 (2.0) | | | 1.000 |
| Transient ischaemic attack, n (%) | 0 (0) | | | 0 (0) | | | 1.000 |
| Myocardial infarction, n (%) | 0 (0) | | | 0 (0) | | | 1.000 |
| Angina pectoris, n (%) | 0 (0) | | | 1 (2.0) | | | 1.000 |
| Congestive heart failure, n (%) | 0 (0) | | | 0 (0) | | | 1.000 |
| Others, n (%) | 23 (47.9) | | | 32 (64.0) | | | 0.109 |
| Complications |  | | |  | | |  |
| Hypertension, n (%) | 30 (62.5) | | | 29 (58.0) | | | 0.649 |
| Dyslipidaemia, n (%) | 38 (79.2) | | | 45 (90.0) | | | 0.137 |
| Hyperuricaemia, n (%) | 3 (6.2) | | | 6 (12.0) | | | 0.487 |
| Arteriosclerosis obliterans, n (%) | 1 (2.1) | | | 1 (2.0) | | | 1.000 |
| Retinopathy, n (%) | 7(14.6) | | | 4 (8.0) | | | 0.302 |
| Nephropathy, n (%) | 26 (54.2) | | | 28 (56.0) | | | 0.855 |
| Neuropathy, n (%) | 9 (18.8) | | | 7 (14.0) | | | 0.525 |
| Hepatic steatosis, n (%) | 24 (50.0) | | | 19 (38.0) | | | 0.231 |
| Chronic kidney disease, n (%) | 0 (0) | | | 0 (0) | | | 1.000 |
| Chronic liver disease, n (%) | 4 (8.3) | | | 5 (10.0) | | | 1.000 |
| Medication |  | | |  | | |  |
| Blood pressure lowering, n (%) | 25 (52.1) | | | 24 (48.0) | | | 0.686 |
| Lipid lowering, n (%) | 27 (56.2) | | | 28 (56.0) | | | 0.980 |
| Anti-coagulant, n (%) | 1 (2.1) | | | 1 (2.0) | | | 1.000 |
| Uric acid lowering, n (%) | 3 (6.2) | | | 2 (4.0) | | | 0.674 |

Data are mean ± SD or n (%) unless otherwise indicated. hs-CRP, high sensitivity C-reactive protein.

Table S2 Details of primary and secondary outcomes

|  |  | Ipragliflozin  n=48 | | | | Metformin  n=50 | | | | Difference between groups | | | | |
| --- | --- | --- | --- | --- | --- | --- | --- | --- | --- | --- | --- | --- | --- | --- |
|  | Weeks | Change from baseline (%) | 95% CI | | | Change from baseline (%) | 95% CI | | | Change from baseline (%) | 95% CI | | | *P*-value |
| Body Composition |  |  |  |  |  |  |  |  |  |  |  |  |  |  |
| Visceral fat area | 24 | -12.06 | -20.55 | , | -3.56 | -3.65 | -11.78 | , | 4.48 | -8.40 | -16.43 | , | -3.38 | 0.040 |
| Subcutaneous fat area | 24 | -7.03 | -11.07 | , | -3.00 | 2.15 | -2.59 | , | 6.89 | -9.18 | -15.34 | , | -3.03 | 0.004 |
| Total fat area | 24 | -7.98 | -12.20 | , | -3.75 | 0.37 | -3.48 | , | 4.22 | -8.35 | -13.98 | , | -2.72 | 0.004 |
| Body weight | 4 | -1.91 | -2.43 | , | -1.39 | -0.11 | -0.52 | , | 0.29 | -1.80 | -2.45 | , | -1.16 | <0.0001 |
|  | 8 | -2.30 | -2.96 | , | -1.63 | -0.27 | -0.77 | , | 0.23 | -2.03 | -2.84 | , | -1.21 | <0.0001 |
|  | 12 | -2.53 | -3.24 | , | -1.81 | -0.77 | -1.38 | , | -0.16 | -1.76 | -2.68 | , | -0.84 | 0.0003 |
|  | 24 | -2.88 | -3.66 | , | -2.11 | -0.74 | -1.62 | , | 0.15 | -2.15 | -3.31 | , | -0.98 | 0.0004 |
| BMI | 4 | -1.91 | -2.43 | , | -1.39 | -0.11 | -0.52 | , | 0.29 | -1.80 | -2.45 | , | -1.16 | <0.0001 |
|  | 8 | -2.30 | -2.96 | , | -1.63 | -0.27 | -0.77 | , | 0.23 | -2.03 | -2.84 | , | -1.21 | <0.0001 |
|  | 12 | -2.53 | -3.24 | , | -1.81 | -0.77 | -1.38 | , | -0.16 | -1.76 | -2.68 | , | -0.84 | 0.0003 |
|  | 24 | -2.88 | -3.66 | , | -2.11 | -0.74 | -1.62 | , | 0.15 | -2.15 | -3.31 | , | -0.98 | 0.0004 |
| Waist circumference | 4 | -0.86 | -1.85 | , | 0.13 | 0.20 | -0.55 | , | 0.94 | -1.06 | -2.27 | , | 0.15 | 0.086 |
|  | 8 | -1.77 | -2.76 | , | -0.78 | 0.01 | -0.99 | , | 1.00 | -1.78 | -3.16 | , | -0.39 | 0.012 |
|  | 12 | -2.22 | -3.26 | , | -1.19 | -0.52 | -1.36 | , | 0.32 | -1.71 | -3.02 | , | -0.40 | 0.011 |
|  | 24 | -2.85 | -3.96 | , | -1.75 | -0.37 | -1.34 | , | 0.59 | -2.48 | -3.92 | , | -1.03 | 0.001 |
| Glycaemic Control |  |  |  |  |  |  |  |  |  |  |  |  |  |  |
| HbA1c | 4 | -4.85 | -6.15 | , | -3.55 | -4.38 | -5.43 | , | -3.34 | -0.47 | -2.10 | , | 1.17 | 0.571 |
|  | 8 | -8.36 | -10.11 | , | -6.61 | -8.34 | -9.76 | , | -6.91 | -0.02 | -2.24 | , | 2.19 | 0.983 |
|  | 12 | -9.24 | -11.26 | , | -7.22 | -10.83 | -12.49 | , | -9.17 | 1.59 | -0.98 | , | 4.16 | 0.222 |
|  | 24 | -8.70 | -11.36 | , | -6.03 | -12.73 | -14.68 | , | -10.78 | 4.03 | 0.79 | , | 7.27 | 0.015 |
| Fasting plasma glucose | 4 | -10.84 | -15.48 | , | -6.20 | -11.98 | -15.53 | , | -8.43 | 1.14 | -4.60 | , | 6.89 | 0.694 |
|  | 8 | -12.84 | -17.59 | , | -8.09 | -15.57 | -18.29 | , | -12.85 | 2.73 | -2.64 | , | 8.10 | 0.315 |
|  | 12 | -13.44 | -17.20 | , | -9.68 | -13.34 | -17.52 | , | -9.16 | -0.10 | -5.66 | , | 5.45 | 0.970 |
|  | 24 | -12.22 | -17.37 | , | -7.08 | -14.15 | -18.07 | , | -10.24 | 1.93 | -4.47 | , | 8.33 | 0.551 |
| Fasting insulin level ^†^ | 12 | -17.65 | -23.53 | , | 0.00 | -11.96 | -23.53 | , | 0.00 | -2.28 | -17.24 | , | 11.39 | 0.745 |
|  | 24 | -20.73 | -31.14 | , | 0.00 | 0.85 | -11.36 | , | 7.84 | -18.56 | -34.20 | , | -2.80 | 0.018 |
| HOMA-beta ^†^ | 24 | 9.05 | -2.42 | , | 35.22 | 26.04 | 14.23 | , | 51.68 | -22.51 | -37.79 | , | -2.18 | 0.029 |
| HOMA-R ^†^ | 24 | -25.25 | -31.87 | , | -4.15 | 0.00 | -14.75 | , | 4.66 | -17.08 | -32.86 | , | -1.91 | 0.024 |
| Blood Lipid Panels |  |  |  |  |  |  |  |  |  |  |  |  |  |  |
| Total cholesterol | 4 | -1.00 | -3.78 | , | 1.77 | -6.65 | -9.73 | , | -3.57 | 5.65 | 1.57 | , | 9.73 | 0.007 |
|  | 8 | -0.22 | -3.39 | , | 2.94 | -6.80 | -10.11 | , | -3.48 | 6.57 | 2.06 | , | 11.09 | 0.005 |
|  | 12 | 1.95 | -1.10 | , | 5.00 | -8.03 | -11.41 | , | -4.65 | 9.98 | 5.51 | , | 14.46 | <0.0001 |
|  | 24 | 1.65 | -1.32 | , | 4.62 | -5.94 | -9.48 | , | -2.41 | 7.60 | 3.07 | , | 12.12 | 0.001 |
| Triglyceride ^†^ | 4 | -16.33 | -25.97 | , | -2.22 | 1.10 | -7.45 | , | 10.77 | -15.75 | -27.53 | , | -1.61 | 0.023 |
|  | 8 | -16.31 | -23.85 | , | -10.53 | -3.98 | -10.33 | , | 5.71 | -15.08 | -26.35 | , | -4.35 | 0.006 |
|  | 12 | -8.81 | -16.18 | , | 3.67 | -4.82 | -15.24 | , | 17.58 | -3.25 | -19.09 | , | 12.09 | 0.604 |
|  | 24 | -14.46 | -19.90 | , | -5.75 | 0.86 | -19.35 | , | 14.95 | -11.49 | -24.92 | , | 4.23 | 0.165 |
| LDL-cholesterol | 4 | 2.34 | -2.26 | , | 6.95 | -7.77 | -12.86 | , | -2.68 | 10.11 | 3.31 | , | 16.91 | 0.004 |
|  | 8 | 1.38 | -3.59 | , | 6.36 | -13.41 | -18.72 | , | -8.10 | 14.79 | 7.61 | , | 21.97 | <0.0001 |
|  | 12 | 3.26 | -1.85 | , | 8.37 | -11.34 | -16.80 | , | -5.87 | 14.59 | 7.22 | , | 21.97 | 0.0002 |
|  | 24 | 3.06 | -1.53 | , | 7.64 | -7.57 | -14.03 | , | -1.11 | 10.63 | 2.81 | , | 18.44 | 0.008 |
| HDL-cholesterol | 4 | 2.18 | -0.45 | , | 4.81 | 0.32 | -2.55 | , | 3.19 | 1.86 | -2.01 | , | 5.73 | 0.342 |
|  | 8 | 5.99 | 2.38 | , | 9.61 | -1.67 | -5.11 | , | 1.77 | 7.66 | 2.74 | , | 12.58 | 0.003 |
|  | 12 | 5.51 | 1.54 | , | 9.48 | 1.59 | -2.17 | , | 5.36 | 3.92 | -1.48 | , | 9.32 | 0.153 |
|  | 24 | 8.74 | 5.12 | , | 12.35 | 1.51 | -2.19 | , | 5.22 | 7.22 | 2.10 | , | 12.34 | 0.006 |
| Other assessments |  |  |  |  |  |  |  |  |  |  |  |  |  |  |
| Systolic blood pressure | 4 | -2.57 | -5.02 | , | -0.12 | -0.88 | -3.32 | , | 1.57 | -1.69 | -5.12 |  | 1.73 | 0.328 |
|  | 8 | -4.27 | -6.46 | , | -2.08 | -1.81 | -3.94 | , | 0.32 | -2.46 | -5.48 | , | 0.56 | 0.109 |
|  | 12 | -3.08 | -5.66 | , | -0.50 | -3.43 | -5.82 | , | -1.04 | 0.35 | -3.12 | , | 3.82 | 0.842 |
|  | 24 | -2.98 | -6.52 | , | 0.56 | -2.19 | -4.60 | , | 0.22 | -0.79 | -4.99 | , | 3.41 | 0.710 |
| Diastolic blood pressure | 4 | -1.19 | -4.46 | , | 2.09 | -0.91 | -4.33 | , | 2.50 | -0.27 | -4.96 | , | 4.41 | 0.908 |
|  | 8 | -4.01 | -7.62 | , | -0.41 | -1.44 | -4.25 | , | 1.37 | -2.57 | -7.05 | , | 1.90 | 0.256 |
|  | 12 | -1.25 | -4.67 | , | 2.18 | -1.80 | -4.79 | , | 1.19 | 0.55 | -3.91 | , | 5.01 | 0.807 |
|  | 24 | -2.93 | -6.89 | , | 1.03 | 0.61 | -3.09 | , | 4.31 | -3.54 | -8.88 | , | 1.81 | 0.192 |
| Adiponectin | 24 | 8.33 | 2.51 | , | 14.14 | 2.20 | -2.29 | , | 6.69 | 6.13 | -1.22 | , | 13.47 | 0.101 |
| hs-CRP ^†^ | 12 | -3.70 | -17.62 | , | 5.00 | -3.16 | -31.25 | , | 12.50 | 1.31 | -23.53 | , | 26.31 | 0.909 |
|  | 24 | -10.18 | -23.32 | , | 10.81 | -18.75 | -38.24 | , | 12.12 | 7.48 | -17.89 | , | 34.62 | 0.590 |

Changes from baseline are shown as means unless otherwise indicated.

^†^: The data were not normally distributed and had outliers; nonparametric analysis (Wilcoxon rank sum test and group difference confidence interval by Hodges-Lehmann estimator) was performed. Change from baseline are shown as median.

hs-CRP, high sensitivity C-reactive protein.

Table S3 Occurrence of adverse events

|  |  | Ipragliflozin  n=49 | | | | Metformin  n=49 | | | | |  |
| --- | --- | --- | --- | --- | --- | --- | --- | --- | --- | --- | --- |
|  | Numbers | Rate (%) | 95% CI | | | Numbers | Rate (%) | 95% CI | | | *P*-value |
| Hypoglycaemia | 2 | 4.1 | 0.5 | , | 14.0 | 2 | 4.1 | 0.5 | , | 14.0 | 1.000 |
| Dehydration | 0 | 0.0 | 0.0 | , | 7.3 | 2 | 4.1 | 0.5 | , | 14.0 | 0.495 |
| Urinary tract infection | 0 | 0.0 | 0.0 | , | 7.3 | 4 | 8.2 | 2.3 | , | 19.6 | 0.117 |
| Drug-induced skin eruption | 2 | 4.1 | 0.5 | , | 14.0 | 0 | 0.0 | 0.0 | , | 7.3 | 0.495 |
| Gastrointestinal disturbances | 7 | 14.3 | 5.9 | , | 27.2 | 26 | 53.1 | 38.3 | , | 67.5 | <0.0001 |
| Diarrhoea | 3 | 6.1 | 1.3 | , | 16.9 | 11 | 22.5 | 11.8 | , | 36.6 | 0.021 |
| Loose stool | 0 | 0.0 | 0.0 | , | 7.3 | 6 | 12.2 | 4.6 | , | 24.8 | 0.027 |
| Constipation | 1 | 2.0 | 0.1 | , | 10.9 | 8 | 16.3 | 7.3 | , | 29.7 | 0.031 |
| Thirst | 7 | 14.3 | 5.9 | , | 27.2 | 0 | 0.0 | 0.0 | , | 7.3 | 0.012 |
| Frequent urination | 14 | 28.6 | 16.6 | , | 43.3 | 1 | 2.0 | 0.1 | , | 10.9 | 0.0003 |

One patient had been allocated to metformin group, however the patient actually had been taking ipragliflozin. Therefore, regarding adverse events, the patient was analysed in the ipragliflozin group.

Table S4 The possible partial correlation between delta change of visceral fat area and other variables

|  | Ipragliflozin |  | Metformin |  | All patients |  |
| --- | --- | --- | --- | --- | --- | --- |
|  | Partial correlation coefficient | *P* value | Partial correlation coefficient | *P* value | Partial correlation coefficient | *P* value |
| Age | -0.17 | 0.293 | -0.03 | 0.866 | -0.08 | 0.453 |
| Waist circumference | -0.07 | 0.661 | -0.19 | 0.232 | -0.12 | 0.249 |
| HbA1c | -0.002 | 0.990 | 0.11 | 0.499 | 0.04 | 0.683 |
| Body weight | 0.07 | 0.642 | 0.10 | 0.522 | 0.09 | 0.399 |
| BMI | -0.19 | 0.298 | -0.01 | 0.973 | -0.05 | 0.684 |
| Duration of diabetes | -0.03 | 0.068 | 0.20 | 0.227 | -0.03 | 0.810 |

# PRIME-V Study Research Organization

**1) Principal Investigator**

　　Koutaro Yokote, MD, PhD

Professor, Department of Clinical Cell Biology and Medicine, Chiba University Graduate School of Medicine

Professor, Department of Medicine, Division of Diabetes, Metabolism and Endocrinology, Chiba University Hospital

1-8-1 Inohana, Chuo-ku, Chiba City, Chiba 260-8670, Japan

TEL：+81-43-226-2092　FAX：+81-43-226-2095

kyokote@faculty.chiba-u.jp

**Steering Committee**

　　Ichiro Tatsuno, MD, PhD

Center of Diabetes, Endocrinology and Metabolism, Toho University Sakura Medical Center

　　Takashi Terano, MD, PhD

Department of Internal Medicine, Chiba Aoba Municipal Hospital

　　Naotake Hashimoto, MD, PhD

Department of Diabetes/Metabolic Endocrinology, Tokyo Women’s Medical University Yachiyo Medical Center

　　Nobuichi Kuribayashi, MD, PhD

Misaki Naika Clinic

　　Daigaku Uchida, MD, PhD

Hotaruno Central Naika

**2) Protocol Committee**

Minoru Takemoto, MD, PhD

Department of Clinical Cell Biology and Medicine, Chiba University Graduate School of Medicine

Kazuki Kobayashi, MD, PhD

Department of Diabetes/Metabolism, Asahi General Hospital

Ko Ishikawa, MD, PhD

Department of Clinical Cell Biology and Medicine, Chiba University Graduate School of Medicine

Masaya Koshizaka, MD, PhD

Department of Clinical Cell Biology and Medicine, Chiba University Graduate School of Medicine

Takahiro Ishikawa, MD, PhD

Department of Clinical Cell Biology and Medicine, Chiba University Graduate School of Medicine

**3) Study Coordinating Investigator**

Ko Ishikawa, MD, PhD

Department of Clinical Cell Biology and Medicine, Chiba University Graduate School of Medicine

**4) Study Coordinating Management Committee**

Koutaro Yokote, MD, PhD

Professor, Department of Clinical Cell Biology and Medicine, Chiba University Graduate School of Medicine

Professor, Department of Medicine, Division of Diabetes, Metabolism and Endocrinology, Chiba University Hospital

Hideki Hanaoka

Clinical Research Center, Chiba University Hospital

Ko Ishikawa, MD, PhD

Department of Clinical Cell Biology and Medicine, Chiba University Graduate School of Medicine

Takatoshi Sato

Clinical Research Center, Chiba University Hospital

**5) Study Coordinating Management Office**

Clinical Research Center, Chiba University Hospital

**6) Auditors**

　　　Increase Co., Ltd. (Tokyo, Japan)

**7) Patient Registration Center / Allocation / Data Management**

　　　Chiba University Clinical Trial Data Center

Mayumi Negishi, Mayumi Matsui, Mayumi Ogawa

The clinical data entry (double data entry), coding, data management, the allocation sequence generation, and reporting will be performed using the data management system ACReSS (Fujitsu, Tokyo, Japan).

**8) Statistical Analysis**

Clinical Research Center, Chiba University Hospital

Sho Takahashi

Department of Global Clinical Research / Biostatistics, Chiba University, Graduate School of Medicine

Kengo Nagashima, Yasunori Sato

**9) Independent Data Monitoring Committee**

Department of Allergy and Collagen Disease, Chiba University Hospital

Shunsuke Furuta

Department of Diabetes/Metabolic Endocrinology, Japanese Red Cross Narita Hospital

Kaori Tachibana

Department of Diabetes/Metabolism, Funabashi Central Hospital

Tsuyoshi Matsumoto

**10) Project Support Organizations**

　　Central Laboratory: LSI Medience Corporation (Tokyo, Japan)

　　Image processing (Contact Research Organization (CRO): Micron Inc. (Tokyo, Japan)

**11) Monitoring**

　　Increase Co., Ltd. (Tokyo, Japan)

**12) Other**

To conduct this study, an agreement was signed between Chiba University and Astellas Pharma Inc. (Tokyo, Japan). Astellas Pharma Inc. funds this study.

**Collaborators**

PRIME-V study group

Study setting: community clinics and academic hospitals in Japan

Each clinical center involved in this study was chosen based on patient availability.

Asahi General Hospital: Hidetaka Yoko, Shunichiro Onishi, Kazuki Kobayashi

Chiba Aoba Municipal Hospital: Takashi Terano, Tomohiko Yoshida, Kyohei Yamamoto, Hanna Deguchi, Tomohiro Ohno

Chiba Chuo Geka Naika: Akina Kobayashi, Ko Ishikawa

Chiba Kaihin Municipal Hospital: Takahiro Ishikawa, Kaneyuki Watanabe

Chiba Rosai Hospital: Masahiro Mimura, Kouichiro Nemoto, Emi Tsuchiya, Yukari Maeda

Chiba University: Ko Ishikawa, Masaya Koshizaka, Kenichi Sakamoto, Masaya Yamaga, Mayumi Shoji, Akiko Hattori, Shintaro Ide, Kana Ide, Akina Kobayashi, Hidetaka Yoko, Takahiro Ishikawa, Yoshiro Maezawa, Minoru Takemoto, Koutaro Yokote, Sho Takahashi, Kengo Nagashima, Yasunori Sato, Takuro Horikoshi

Funabashi Central Hospital: Hidetaka Yoko, Masaya Koshizaka

Funabashi Municipal Medical Center: Hideaki Iwaoka, Tatsushi Shimoyama, Syunsuke Nakamura

Hotaruno Central Naika: Daigaku Uchida, Susumu Nakamura

Inage Hospital: Minoru Takemoto, Harukiyo Kawamura, Kenichi Sakamoto

Izumi Chuo Hospital: Minoru Takemoto.

Kimitsu Chuo Hospital: Ryouichi Ishibashi, Tomoko Takiguchi, Kenji Takeda.

National Hospital Organization Chiba Medical Center: Fumio Shimada, Hirotake Tokuyama, Tetsuya Okazaki, Kenchi Yui, Emi Ohara.

Kujyukuri Home Hospital: Ko Ishikawa.

Kouyukai Memorial Hospital: Akiko Hattori, Masaya Yamaga.

Sannou Hospital: Ryouta Shimousa.

Seirei Sakura Citizen Hospital: Kana Ide, Mayumi Shoji, Ryouichi Ishibashi.

Sousa Citizen Hospital: Yusuke Baba, Masaya Yamaga, Ryoichi Ishibashi.

Tamura Memorial Hospital: Kenichi Sakamoto, Shintaro Ide.

Toho University Sakura Medical Center: Ichiro Tatsuno, Atsuto Saiki, Yasuhiro Watanabe.

Tokuyama Clinic: Takahiko Tokuyama.

Tokyo Women’s Medical University Yachiyo Medical Center: Jun Ogino, Naotake Hashimoto, Chihiro Yoneda, Kana Tajima.

**The primary endpoint evaluation committee**

　Diagnostic Radiology and Radiation Oncology, Chiba University Graduate School of Medicine

Takuro Horikoshi

　Department of Radiology, Sannou Hospital

Ryouta Shimofusa

**The Institutional Review Boards (IRBs), Ethics Review Committees, or Ethics Committees**

Chiba University Hospital (ID number: G26009), Asahi General Hospital (ID number: 2014091602), National Hospital Organization Chiba Medical Centre, Seirei Sakura Citizen Hospital, Chiba Rosai Hospital (ID number: 26-21), Toho University Sakura Medical Centre (ID number: 2014-077), Tokyo Women’s Medical University Yachiyo Medical Centre (ID number: 150303), Chiba Aoba Municipal Hospital, Kimitsu Chuo Hospital, Funabashi Central Hospital (ID number: H27-1), and Chiba Kaihin Municipal Hospital. In other facilities, approval was provided at Chiba University Hospital (acting as the centralised IRB)
